# Spatio-Temporal Epidemiology of the Spread of African Swine Fever in Wild Boar and the Role of Environmental Factors in South Korea

**DOI:** 10.3390/v14122779

**Published:** 2022-12-13

**Authors:** Satoshi Ito, Jaime Bosch, Hyunkyu Jeong, Cecilia Aguilar-Vega, Jonghoon Park, Marta Martínez-Avilés, Jose Manuel Sánchez-Vizcaíno

**Affiliations:** 1 VISAVET Health Surveillance Center, Complutense University of Madrid, 28040 Madrid, Spain; 2 Department of Animal Health, Faculty of Veterinary, Complutense University of Madrid, 28040 Madrid, Spain; 3 Dodram Pig Research Center, Daejeon 35377, Republic of Korea; 4 Independent Scholar, Daejeon 35377, Republic of Korea; 5 Animal Health Research Centre (CISA-INIA/CSIC), 28040 Madrid, Spain

**Keywords:** African swine fever, spatio-temporal epidemiology, wild boar, basic reproduction number (R_0_), Asia, South Korea, regression model, environmental factor

## Abstract

Since the first confirmation of African swine fever (ASF) in domestic pig farms in South Korea in September 2019, ASF continues to expand and most notifications have been reported in wild boar populations. In this study, we first performed a spatio-temporal cluster analysis to understand ASF spread in wild boar. Secondly, generalized linear logistic regression (GLLR) model analysis was performed to identify environmental factors contributing to cluster formation. In the meantime, the basic reproduction number (R_0_) for each cluster was estimated to understand the growth of the epidemic. The cluster analysis resulted in the detection of 17 spatio-temporal clusters. The GLLR model analysis identified factors influencing cluster formation and indicated the possibility of estimating ASF epidemic areas based on environmental conditions. In a scenario only considering direct transmission among wild boar, R_0_ ranged from 1.01 to 1.5 with an average of 1.10, while, in another scenario including indirect transmission via an infected carcass, R_0_ ranged from 1.03 to 4.38 with an average of 1.56. We identified factors influencing ASF expansion based on spatio-temporal clusters. The results obtained would be useful for selecting priority areas for ASF control and would greatly assist in identifying efficient vaccination areas in the future.

## 1. Introduction

African swine fever (ASF), caused by the ASF virus (ASFV), is one of the most important transboundary animal diseases. ASFV is a double-stranded DNA virus of about 170–190 kbp and belongs to the family Asfaviridae. Pigs, including wild boar, are generally regarded as susceptible hosts. The clinical signs exhibited by infected individuals vary and are classified into four main stages based on clinical presentations and pathological lesions: peracute, acute, subacute, and chronic [1]. Susceptible animals can become infected via direct contact with infected individuals [2,3]. In addition, indirect contact with ASFV-contaminated material may play an important role in the spread of the virus over long distances [4]. One of the main characteristics of ASFV is its high environmental resistance. It is well known that the virus can maintain its infectivity for a long time under various conditions. The virus is shed in large quantities in the blood where the virus can survive for 15 weeks at room temperature, months at 4 °C, and indefinitely when frozen [5]. In the case of raw meat, it can survive for more than three months in meat and offal [6]. Feces and urine are also infectious; the half-life of the virus in urine is 15 days depending on the environmental temperature [7], and in feces, it is reported to be 5–8 days, but viral DNA can be detected for up to 2–4 years [7]. Infected carcasses could contaminate soil and be exposed to other wild boar, which heightens the risk of disease spread [4,5].

**Figure 1:**
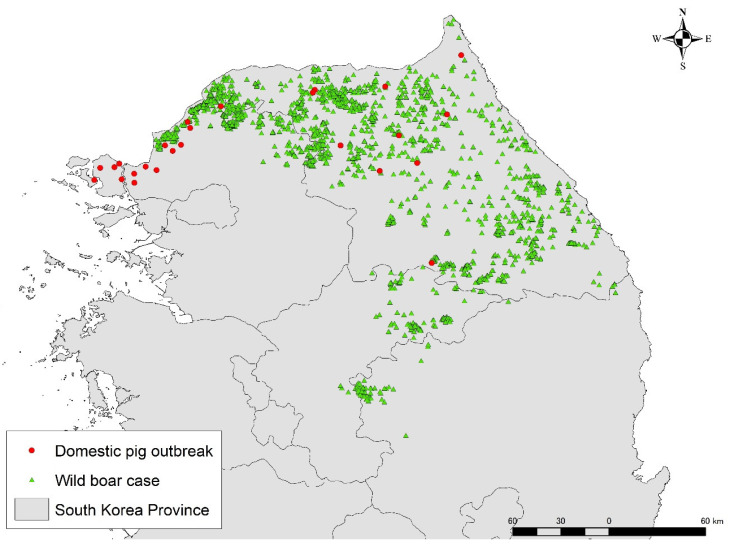
African swine fever notifications in South Korea as of the end of September 2022. The map was depicted in ArcGIS software version 10.8.1 based on the given data. Red circle indicates outbreaks in domestic pig farms, while green triangles represent wild boar cases.

In Asia, since the first ASF notification was reported in China in 2018, outbreaks have been confirmed in 17 countries (China, Mongolia, Vietnam, Cambodia, North Korea, Laos, Myanmar, the Philippines, South Korea, East Timor, Indonesia, Papua New Guinea, India, Malaysia, Bhutan, Thailand, and Nepal) as of the end of September 2022 [8]. Most of these ASF-affected countries reported the disease in domestic pig sectors, which was presumably due to inadequate biosecurity measures and illegal trade of infected pigs, as well as the transport of contaminated pork products [9]. Contrary to this, the majority of notifications in South Korea have been reported in wild boar populations [10]. The first ASF outbreak was confirmed in September 2019 at a pig farm in the northwestern part of the country [11]. About half a month later, the first ASF case in wild boar was reported along the border with North Korea, about 33.5 km distant from the first reported ASF-outbreak pig farm [11]. Immediately after confirmation of the wild boar case, fencing around the case-reported area was implemented to contain further spread of ASF infection in wild boar [12]. Since then, the government has been actively working on population control by searching for and hunting wild boar, as well as installing fences around infected areas to prevent the spread of infection within the wild boar population [12]. In South Korea, government-led search teams are organized nationwide, by region, to search for wild boar on a constant basis [11,13]. The search teams are composed of a variety of people, including civilians, hunters, and military personnel, and the government facilitates the search by offering a bounty for the discovery of wild boar. Search areas are concentrated in and around fenced infected areas, and when a carcass is found, they are obligated to report it to the municipality. These carcasses are to be buried at the place of discovery after being sampled. Recently, drones have been introduced to search inaccessible areas and detection dogs are used to search for carcasses [14,15]. However, the number of infected wild boar continues to increase while expanding the infected areas (Gangwon-do, Gyeonggi-do, Chungcheongbuk-do, and Gyeongsangbuk-do), with 2661 cases reported as of the end of September 2022 [16]. During this period, strict management of biosecurity measures has also been promoted at pig farms to prevent the virus from entering the farms [12]. However, ASF outbreaks on pig farms have occurred sporadically each year, with 27 outbreaks reported as of the end of September 2022 [16] (Figure 1).

To control ASF, it is crucial to understand the epidemiology of the disease and determine the factors that influence its spread. This requires a holistic understanding of the natural, geographical, and socio-cultural background of the disease, as well as ecological knowledge. The population density of wild boar is one of the important factors contributing to disease expansion, while related information is limited in most cases. In South Korea, information regarding wild boar population density is disclosed by the government [17] and relevant information is also available in some areas [18]. However, on a national scale, detailed data on habitat distribution and density are largely unknown [19]. 

The objective of this study was to elucidate the spread of ASF in wild boar populations by spatio-temporal epidemiological analysis and to identify the influence of environmental factors on the epidemic status. Furthermore, the evolution of ASF in wild boar populations was elucidated by determining basic reproduction numbers in infected populations.

## 2. Materials and Methods

Based on the geographic and temporal data obtained regarding ASFV PCR-positive wild boar, a spatio-temporal cluster analysis was performed. Then, generalized linear logistic regression (GLLR) models were used to analyze environmental factors that may contribute to cluster formation. In the meantime, we estimated the basic reproduction number (R_0_) within each cluster to understand the growth of the epidemic in the wild boar population.

### 2.1. Epidemiological Data Preparation

Epidemiological information about ASFV PCR-positive wild boar (geographic coordinates and notification date) reported between 16 September 2019 and 1 September 2022 was provided by the Dodram pig research center, South Korea [20].

### 2.2. Identification of Spatio-Temporal Clusters

A space-time permutation technique was applied to identify the spatial and temporal concentrations of ASF cases in wild boar during the study period. Three key parameters had to be determined for the implementation of the model: the maximum size of the temporal and spatial clusters and the time aggregation. A previous study indicated that ASF cases in wild boar relate to the season [21]. There are indeed seasonal variations in the ASF epidemic trend in South Korea [22]. Thus, we set a maximum window size of 90 days. The minimum time aggregation was set as seven days to consider the within-week variability of surveillance activities [23]. To define the maximum size of the spatial clusters, a multi-distance spatial cluster analysis tool based on Ripley’s K function was applied on ArcGIS software version 10.8.1 (ESRI, Redlands, CA, USA) following the manufacturer’s tool guidance [24]. For the analysis of the spatial pattern of ASF cases in wild boar, observed K values were compared to the expected K values of a completely random spatial distribution of ASF detection with 999 simulations, equivalent to a confidence level of 99.9%. Diff K contains the observed K values minus the expected K values. In this study, the expected K values giving the highest diff K were set as the upper limit on the geographical size of the cluster. A Monte Carlo process was implemented using 999 replications to test for the presence of candidate clusters (*p* ≤ 0.05). Analyses were performed in SaTScan software v9.6 (Kulldorff, Boston, MA, USA) [25].

### 2.3. Identification of Environmental Factors Contributing to Spatio-Temporal Cluster Formation Based on GLLR Model

The density of wild boar is presumed to be one of the factors contributing to ASF spread, but the associated information is usually limited. Thus, an attempt was made to estimate areas with a high number of ASF cases in wild boar based on environmental factors. To examine the influence of environmental factors on spatio-temporal cluster formation, a generalized linear logistic regression (GLLR) model was developed with a binary response (logit link). A binary number was selected for the response variable, whether the wild boar case formed a spatio-temporal cluster or not (1 for in-cluster and 0 for out-of-cluster). Various environmental variables were selected as explanatory variables based on previous studies, knowledge, and experience: elevation, distance from road (roadway and sidewalk), wild boar distribution index, travel time to major cities, soil moisture, temperature seasonality (bioclim 4), temperature annual range (bioclim 7), precipitation seasonality (bioclim 15), precipitation of warmest quarter (bioclim 18), and precipitation of coldest quarter (bioclim 19). Previous studies identified Elevation as a factor that affects wild boar habitat [26]. The data were obtained from the SRTM 90 m DEM Digital Elevation Database [27]. Some infected areas may not have adequate surveillance due to the precipitous mountain ranges, and thus the distance from the nearest road to the wild boar case was included as a variable. Road maps (roadway and sidewalk) were retrieved from the Korea National Spatial Data Infrastructure Portal [28] and distance from road was then calculated with the “Near” tool in ArcGIS 10.8.1 [29]. The wild boar distribution index was generated by multiplying the seasonal normalized difference vegetation index (NDVI) [30] and quality of available habitat (QAH) map for wild boar developed by Bosch et al., [31]. The QAH map is a tool that quantifies the optimal habitat for wild boar on seven levels (0, 0.1, 0.5, 1, 1.5, 1.75, 2) [32]. NDVI is an index measuring the quantity, quality and development of vegetation and is thus often incorporated in the species distribution model as one of the predictors [33]. Travel time to major cities was defined as the travel time to a location of interest using land (road/off-road) or water (navigable river, lake and ocean) from places with a population of 50,000 people or more [34]. As such, it can be assumed as an indirect indicator of landscapes such as urban and rural areas. Data were available from the Forest Resources and Carbon Emissions database managed by the Directorate-General for Joint Research Centre [34]. As ASF cases in wild boar have been found both in natural areas and in agroforestry and agro-urban areas, the variable was used to assess the impact of different landscapes. Soil moisture is considered one of the variables that determines surface ecosystem health and stress [35] and may be indirectly related to vegetation cover and food resource availability as it affects a range of soil and plant dynamics. The soil moisture data were retrieved from the Center for Sustainability and the Global Environment database [36]. The association between ASF cases in wild boar and climatic factors has been well discussed and used in previous studies [37]. From these, we selected several factors based on our knowledge and experience. Data on climatic factors were obtained from WorldClim; temperature seasonality (bioclim 4), temperature annual range (bioclim 7), precipitation seasonality (bioclim 15), precipitation of warmest quarter (bioclim 18), and precipitation of coldest quarter (bioclim 19) [38].

Since no simple linear relationship was found between response and explanatory variables, all explanatory variables were categorized [39]. Variables were equally divided such that the sample size for each category was as equal as possible. A single regression analysis was performed to examine the relationship between each explanatory and response variable. Variables with a *p*-value greater than 0.15 were excluded at this stage. Likelihood ratio tests were used to compare models with the explanatory variables of interest to null models with no explanatory variables. The null hypothesis was “of the two models compared, the more complex model has the same or less goodness of fit to the data than the less complex model”. If the *p*-value was very small (less than *p* = 0.05), the null hypothesis was rejected, and the variable of interest was selected as a candidate for inclusion in the multivariate model. This step was performed with the “lrtest” function of the “lmtest” package in the R programming environment [40].

The GLLR model was developed using the “glmer” function in the “lme4” package [41] with the explanatory variables selected in the univariate analysis as fixed effects and the season (Spring: Mar–May; Summer: Jun–Aug; Autumn: Sep–Nov and Winter: Dec–Feb) as random effects. Variance inflation factor (VIF) analysis was performed using the “vif” function in the “car” package [42] to account for multicollinearity among the explanatory variables. Here, variables with a VIF > 3 were eliminated. The backward elimination and forward selection procedures were then used to select the best model (criterion: *p*-value ≤ 0.05). All analyses were performed under the R programming environment.

The le Cessie-van Houwelingen normal test, proposing a class of tests based on smoothed residuals, was used to determine the goodness of fit of the model (criterion: *p*-value ≤ 0.05) [43,44]. A receiver operating characteristic (ROC) curve was complimentarily drawn and subsequently area under the curve (AUC) was calculated. An AUC of 1 was the most optimal and a value of 0.5 implied no better than a random model [45].

### 2.4. Estimation of Basic Reproduction Number (R_0_) in the Spatio-Temporal Cluster

The basic reproduction number (R_0_) is a well-known epidemiological indicator of the contagiousness of an infectious disease [46]. When an infectious agent enters a susceptible population, the average number of secondary infectious agents that reproduce during the entire infectious period is defined as R_0_. If R_0_ > 1, the epidemic will expand, but if R_0_ < 1, the epidemic will spontaneously disappear. The basic mathematical model for determining R_0_ is called the SIR model, which divides the population into three compartments (susceptible, infectious, and removed/recovered) according to the stage of infection and models the temporal changes in infection-related status [47]. However, when determining R_0_ for wildlife infections, the number of susceptible populations is usually unknown. To address these issues, an attempt was made to assume that cases grow at an exponential rate during the early stages of the epidemic and compute R_0_ at this stage as a function of doubling time and infectious period [47,48]. Estimation of R_0_ for the ASF epidemic in wild boar populations has been performed several times in combination with spatio-temporal cluster analysis [49,50,51]. Here, we followed previous studies to calculate R_0_ within each spatio-temporal cluster detected.

Initially, case data assigned to each spatio-temporal cluster was log-transformed and fitted to a linear model to evaluate the fit of the data sets to an exponential distribution. Adjusted R-squared values were obtained to assess model fit for each subset. These steps were performed with Microsoft Excel. According to Anderson et al. [47], given the assumption that outbreaks increase exponentially in the initial epidemic, the number of cases λ(*t*) at time *t* can be estimated from the initial number of cases λ(0) and the growth rate Λ using the following formula.

$$\lambda \;\left(t\right)=\lambda \;\left(𝟎\right)e^{\left(\Lambda t\right)}$$

Growth rate Λ can then be expressed as follows.

$$\Lambda =\left(R_{0}-𝟏\right)/D$$

Citing previous literature [48,50], R_0_ can be obtained by the following equation based on the above equations, assuming the initial number of cases is 1.

R_0_ = 1 + (*D*/*t*_d_) ln2

where *D* indicates the infectious period, and *t*_d_ is the time taken for the number of infections to double. While the value of *t*_d_ could be calculated from the epidemic curve within a cluster [48,50], *D* had to be determined based on virological and epidemiological insights. In this study, we considered “wild boar–habitat cycle”, which is characterized by both direct transmission between infected and susceptible wild boar and indirect transmission via carcasses in the habitat [52]. The transmission cycle may differ depending on the infected area and the timing of the occurrence. Some areas may be well-surveilled with most infected carcasses being quickly detected and properly removed. On the other hand, there may be areas where surveillance is inadequate and infected carcasses are left for long periods of time due to geographical or weather conditions, or low priority of the surveillance, etc. In the real situation, we believed these scenarios to be mixed, depending on location and time period. Unfortunately, there was no information available about this. Alternatively, we have indicated two separate scenarios. The first only considers direct transmission between wild boar. This scenario assumes that most ASF-infected carcasses were found by surveillance or decomposed quickly, such that carcasses were not included in the transmission cycle. According to the previous study, the infectious period of a highly virulent ASFV strain is between 2 and 9 days, assuming direct transmission between wild boar [53]. Assuming that the prevalent strain in South Korea is also highly virulent [54], the minimum and maximum *D* was set as 2 and 9 days, respectively. On the other hand, positing the second scenario, wherein infected carcasses were not promptly removed but remained, indirect transmission via these carcasses may play an important role in the spread of ASF in the wild boar population [55]. In this case, the infectious period was considered to be highly dependent on environmental factors, such as temperature, vegetation, and sunlight [56,57]. Here, this was taken as the period between infection and skeletonization of wild boar (when the carcass decomposes into skin and bones), referring to a previous study conducted in South Korea on seasonal patterns of carcass decomposition [58]. In summer, it took 7–12 days for fresh carcasses to skeletonize, while in winter this required 11–51 days. Thus, the minimum and maximum *D* in each cluster was set considering the influence of the season. As information regarding spring and autumn was not available, the average *D* for winter and summer was applied. Hence, minimum, and maximum *Ds* in spring, summer, autumn, and winter were set as 11–40.5, 9–21, 11–40.5, and 13–60 days, respectively. Since no further information was available, we assumed a uniform distribution and performed 10,000 iterations to compute R_0_ in the R programming environment.

## 3. Results

Epidemiological information was obtained for a total of 2658 ASF wild boar cases confirmed by a PCR test in the government laboratory.

### 3.1. Identification of Spatio-Temporal Clusters

**Figure 2:**
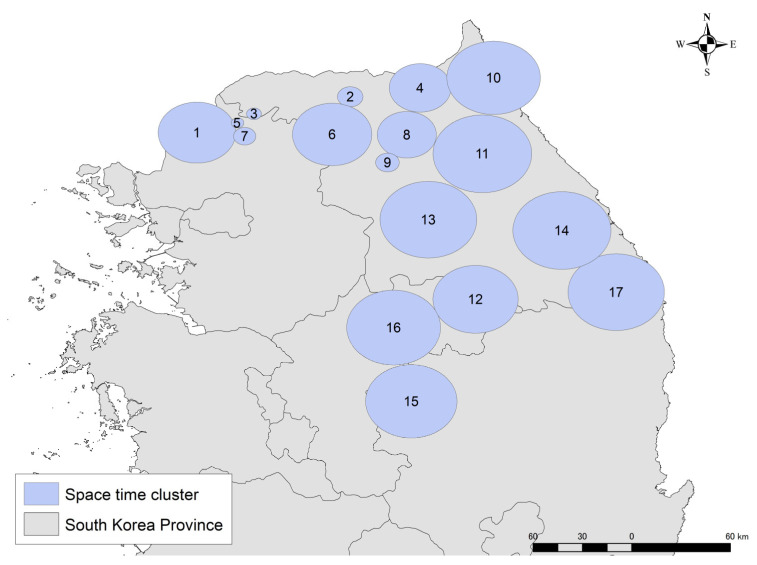
Locations of the significant space-time clusters of African swine fever (ASF) cases in wild boar. The spatio-temporal clusters are depicted by light blue circles, and the clusters are ordered chronologically.

The upper limit of the geographical size of the cluster was set as 23.7 km, which resulted from multi-distance spatial cluster analysis. A total of 17 significant spatio-temporal clusters were detected (*p* ≤ 0.05) (Figure 2). No spatio-temporal clusters were detected in 2019. Many clusters were detected in 2020–2021, mainly in the northern part of the country. These were found repeatedly in the same areas in different periods. Multiple clusters detected from the end of 2021 to July 2022 were located in more southern areas (Table 1). This suggested that the epidemic center was gradually moving southward. Classifying each season as spring (March to May), summer (June to August), autumn (September to November), and winter (December to February), most clusters were found in winter (*n* = 9), followed by summer (*n* = 4), spring (*n* = 3) and autumn (*n* = 1).

### 3.2. Identification of Environmental Factors Contributing to Spatio-Temporal Cluster Formation Based on a GLLR Model

As a result of univariate analysis, the precipitation seasonality (bioclim 15) variable was excluded from the candidate explanatory variables at this stage (*p* > 0.15) (Table 2). A subsequent likelihood ratio test against the null model excluded the temperature seasonality (bioclim 4) variable and selected the remaining variables as candidates for inclusion in the GLLR model (*p* ≤ 0.05).

Since all explanatory variables had a VIF < 3, no variables were eliminated in terms of multicollinearity. After the backward elimination and forward selection procedure, the GLLR model was finally constructed with variables of elevation, distance from road, wild boar distribution index, travel time to major cities, soil moisture, temperature annual range (bioclim 7), precipitation of warmest quarter (bioclim 18), and precipitation of coldest quarter (bioclim 19) as fixed effects and season as random effects. Elevation and travel time to major cities were positively related to the response variables, but the wild boar distribution index related differently to the response variable depending on the grade. The remaining variables were negatively correlated with the response variable (Table 3).

**Figure 3:**
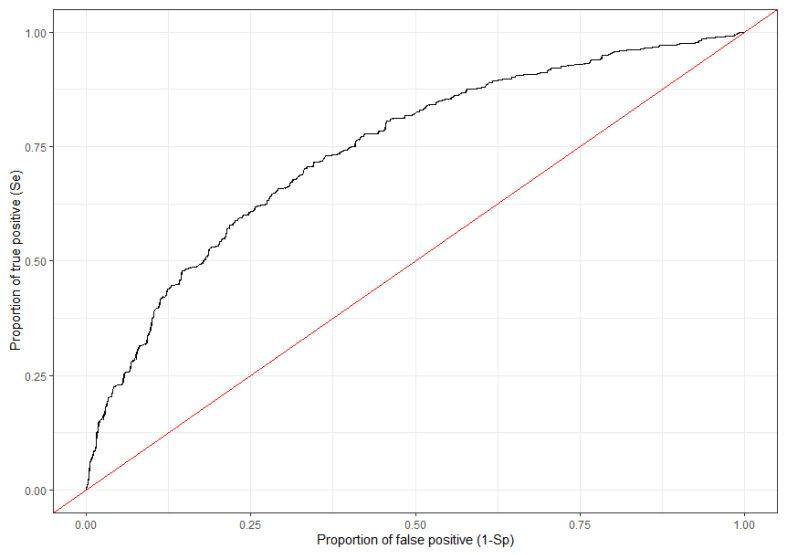
ROC curve (receiver operating characteristic curve) for the Generalized linear logistic regression model was plotted with the true positive ratio (Se) against the false positive ratio (1-Sp), where Se represented the *y*-axis and 1-Sp the *x*-axis.

The null hypothesis of the le Cessie-van Houwelingen normal test is “no lack of fit”. The results obtained presented a *p*-value of 0.199, thus indicating the validity of our model. The ROC curve was drawn based on the multivariate model obtained and the AUC was calculated to be 0.743 (Figure 3).

### 3.3. Estimation of Basic Reproduction Number (R_0_) in the Spatio-Temporal Cluster

In Scenario 1, which only assumed direct transmission among wild boar, R_0_ ranged from 1.01 to 1.51, with an average of 1.10. In scenario 2, which also included indirect transmission via infected carcass, R_0_ ranged from 1.03 to 4.38, with an average of 1.56 (Table 1). The adjusted R-square value was generally higher. Of the 17 clusters, 13 were over 0.9 and two over 0.8. The remainder were 0.7 and 0.65.

## 4. Discussion

The current approach was implemented to understand the evolution of ASF in wild boar populations and the influence of environmental factors on cluster formation. The results of the spatio-temporal cluster analysis indicated that, while clusters were repeatedly detected at different times in the same area, some clusters were detected in more southern areas in 2022. Of the 17 clusters detected, nine were identified in winter, which is consistent with the seasonal variation in the number of ASF case reports among wild boar in South Korea (the number of reports peaks in winter, with the lowest number in summer). It is assumed that finding animals is more difficult in summer because of the thick foliage on the trees, whereas, in winter, the leaves fall off the trees making it easier to detect wild boar. Active surveillance searching for wild boar is regularly conducted throughout the year. However, these natural phenomena might contribute to the seasonality trend in the case of ASF in wild boar.

Spatio-temporal cluster analysis depends on spatio-temporal information regarding wild boar cases. However, the multivariate analysis results obtained suggested the possibility of predicting ASF hotspots in wild boar based on environmental conditions and timing of occurrence in South Korea (AUC: 0.743). In general, an AUC of 0.7 or higher is considered “good” accuracy, and 0.8 or higher is considered “excellent” accuracy. In countries where disease outbreaks are endemic, prioritization of control areas is not a straightforward task. Disease control can be even more complex, especially when target animal populations fluctuate widely and have large distribution areas. For the purpose of estimating the distribution area and population density of wild boar, camera traps are commonly used to observe the behavior patterns of wild boar. While highly reliable data can be obtained, it is difficult to implement over a wide area from the standpoint of cost and time. In this respect, if we can identify areas where outbreaks are likely to be clustered based on environmental conditions, more efficient countermeasures can be taken.

Multivariate analysis showed that the explanatory variables elevation, travel time to major cities and precipitation of warmest quarter (bioclim 18) were positively correlated with spatio-temporal cluster formation as a response variable. Negative correlations were observed for distance from road, soil moisture, temperature annual range (bioclim 7), and precipitation of coldest quarter (bioclim 19). The wild boar distribution index showed a positive correlation in grade V but a negative correlation in grade IV. The affected area extends around the Taebaek Mountains that traverse the eastern part of the Korean Peninsula. Considering that the lower elevation areas are human habitation areas and higher elevation areas are mountainous but human-accessible areas, it is understandable that Grades III and IV have a strong positive correlation with the response variable. We hypothesized that differences in the behavioral patterns of wild boar in forested and near-urban areas [59] might have some influence on the spread of infection, but our study did not provide sufficient results to support this assumption. A positive correlation with the variable of travel time to major cities was found in grade IV, which indicated that ASF cases were concentrated in remote forest areas. This might be because wild boar are cautious animals, and their preferred habitat areas are resource-rich forested areas [60]. The wild boar distribution index is a quantified measure of habitat quality for wild boar and was used to indirectly describe their abundance. Here, grade V showed a significant positive correlation with the response variable. It has been pointed out that wild boar seek out their last resting place after infection with ASF [61], and the Grade V area is considered to be such an area. Assuming that direct contact between infected carcasses and wild boar could occur [62], this would explain the significant effect on cluster formation. The present results for the variables temperature annual range (bioclim 7) and precipitation of warmest quarter (bioclim 18) were consistent with findings from previous studies. A negative correlation between wild boar density and temperature annual range (bioclim 7) had been found in northeastern Europe [63]. The significant positive effect of increased precipitation in the warm season on cluster formation (grades II and III) can be explained by observations from a past study in Sweden and current problems of fence installation in South Korea. Increased precipitation during the warm season increases wild boar activity [64], thus potentially increasing their chances of encountering infected animals or carcasses. In addition to ecological factors for wild boar, anthropogenic factors may have contributed to this outcome. In South Korea, the problem of installed fences collapsing due to heavy rains in the summer has been frequently reported in many areas [65]. During the fence repair period, wild boar can freely move in and out of the fenced area through the damaged sites, hence the opportunities for contact between wild boar may increase as their range of activity expands. Soil moisture is “the total amount of water, including water vapor, in unsaturated soil” and represents the surface water present in soil pores [66]. The negative correlation between soil moisture and wild boar ASF cluster formation presented here cannot be easily explained. One possibility is that the results may suggest optimal environments for certain plants and organisms, which may be indirectly related to the ecology of wild boar. In this respect, further research is needed.

The correlation between environmental factors and spatio-temporal cluster formation has been discussed. However, it should be noted that the topography of South Korea is mountainous and steep, which may pose limitations for surveillance activities in infected areas. The negative correlation between the variable distance from road grade IV and cluster formation suggests that areas relatively far from roads are less frequently reported due to difficult access. Similarly, winter precipitation (bioclim 19) in northeastern South Korea can be considered snow cover, and areas with higher precipitation can be considered areas with harder-to-reach surveillance. In a country such as South Korea, where ASF outbreaks are endemic to some areas while expanding in others, the selection of surveillance areas is challenging. In terms of economic losses, protecting pig farmers from ASF would be the top priority. However, attention should always be paid to the possibility that the disease could be maintained in surveillance inaccessible areas.

In recent years, several studies have been conducted to compute R_0_ in spatio-temporal clusters with various approaches [49,50,51]. The estimation of the infectious period (*D*) is key to the computation of R_0_, but conditions probably differ depending on the virulence of the virus, susceptibility of the host, and the mode of transmission. Here, we considered scenario 1, which only considered direct transmission, and scenario 2, which also included indirect transmission via infected carcasses. While there appeared to be no seasonal difference in R_0_ between clusters in scenario 1, the R_0_ of clusters observed in winter was generally higher than that of other seasons in scenario 2. In winter, the decomposition of infected carcasses takes more time, resulting in a longer exposure period to other wild boar. The shorter case doubling period in winter compared with other seasons is logical when assuming that indirect transmission plays an important role. Perhaps direct transmission is the primary mode of transmission in the summer, when carcasses decompose more quickly, and indirect transmission plays a more important role in winter. Since sufficient evidence does not exist to support this, further investigation is required. The association between surveillance bias and increasing numbers of winter cases was discussed earlier, but these ecological factors may also contribute.

In this study, we attempted to estimate the hot spot areas of ASF-positive wild boar from environmental conditions. Moreover, we analyzed the evolution of the disease within the clusters, accounting for the mode of transmission and seasonality. Since information on the distribution and abundance of wild boar is generally scarce, this method is useful for the rapid estimation of high outbreak areas based on available information. In situations where the disease is spreading widely, priority control areas need to be selected. This tool will also play an important role in selecting vaccination areas when a vaccine against ASF for wild boar is commercialized in the future.

Information on ASF-negative wild boar was not available in the current study. Therefore, the analysis was conducted based on the location of positive cases. In South Korea, active surveillance has been promoted since the early stages of the outbreak and population reduction measures have been implemented [67]. If further information were accessible, more precise assessments of epidemic status, identification of risk areas, and estimates of future spread could be achieved.

Recently, the emergence of lower virulent ASFV has been reported in China [68,69]. These are reported to be characterized by a long incubation period, unclear clinical signs, and low lethality [68,69]. At present, there is no information on the severity of the disease in wild boar. In South Korea, where the majority of positive cases are found as carcasses, it will be more challenging to control the disease in the event that these viruses are introduced into the wild boar population. Thus, early detection will be the key [70]. In the end, we believe that this article provides important knowledge on the status of ASF in South Korea and other Asian countries.

## Figures and Tables

**Table 1:** Detailed information regarding each spatio-temporal cluster and R_0_. Scenario 1 considers direct transmission between wild boar, while Scenario 2 includes indirect transmission via infected carcasses. The season containing more days was assigned.

Cluster No.	Number of Cases	Start Date	End Date	Season	Case Doubling Time (Day)	Adjusted R-Square	Scenario 1 R_0_ (D_min_, _max_ = 2, 9)	Confidence Interval (95%)	Scenario 2 R_0_	Confidence Interval (95%)	Scenario 2 *D* (min–max)
1	165	2020/1/9	2020/4/1	Winter	37.5	0.95	1.10	1.04–1.16	1.66	1.27–2.09	13–60
2	159	2020/1/16	2020/4/8	Winter	35.7	0.93	1.11	1.04–1.17	1.72	1.28–2.14	13–60
3	43	2020/3/12	2020/5/13	Spring	63.6	0.93	1.06	1.02–1.1	1.28	1.13–1.43	11–40.5
4	32	2020/8/13	2020/11/4	Autumn	117.5	0.96	1.03	1.01–1.05	1.15	1.07–1.23	11–40.5
5	18	2020/12/3	2020/12/16	Winter	12.1	0.83	1.33	1.13–1.51	3.06	1.81–4.38	13–60
6	135	2021/1/7	2021/3/17	Winter	32.1	0.93	1.12	1.05–1.19	1.82	1.3–2.27	13–60
7	66	2021/1/21	2021/4/14	Winter	55.0	0.96	1.07	1.03–1.11	1.45	1.18–1.74	13–60
8	42	2021/2/11	2021/5/5	Spring	73.0	0.96	1.05	1.02–1.08	1.25	1.11–1.38	11–40.5
9	9	2021/6/17	2021/7/21	Summer	105.0	0.97	1.04	1.01–1.06	1.10	1.06–1.14	9–21
10	11	2021/7/8	2021/8/25	Summer	138.6	0.95	1.03	1.01–1.04	1.07	1.05–1.1	9–21
11	62	2021/7/22	2021/9/15	Summer	36.3	0.92	1.11	1.04–1.17	1.29	1.18–1.39	9–21
12	99	2021/11/18	2022/1/5	Winter	29.1	0.65	1.13	1.05–1.21	1.84	1.34–2.4	13–60
13	50	2021/12/23	2022/2/16	Winter	51.0	0.93	1.07	1.03–1.12	1.49	1.2–1.8	13–60
14	189	2022/1/6	2022/3/30	Winter	36.1	0.94	1.11	1.04–1.17	1.70	1.28–2.13	13–60
15	95	2022/2/3	2022/4/27	Spring	56.4	0.95	1.07	1.03–1.11	1.31	1.14–1.49	11–40.5
16	35	2022/2/17	2022/3/9	Winter	19.7	0.80	1.20	1.08–1.31	2.30	1.5–3.07	13–60
17	5	2022/6/9	2022/7/6	Summer	231.0	0.70	1.02	1.01–1.03	1.05	1.03–1.06	9–21

**Table 2:** Results of univariate analysis: the relationship between response and explanatory variables. Each explanatory variable was categorized (equally divided from grade I to a maximum of V by descending value). Variables with *p*-values less than 0.15 (in bold) were advanced to the next step.

Variables	Grade (Threshold)	Total Samples (n = 2578)	Coefficients	Standard Errors	*p*-Value
Elevation	Grade I (232)	648	-	-	-
	**Grade II (352)**	**649**	**−0.185**	**0.079**	**0.099**
	**Grade III (490)**	**640**	**0.261**	**0.112**	**0.020**
	Grade IV (1157)	641	0.082	0.112	0.459
Distance from road (roadway and sidewalk)	Grade I (276.2)	645	-	-	-
	Grade II (602)	644	−0.009	0.111	0.933
	Grade III (1171.5)	644	−0.072	0.112	0.521
	**Grade IV (6887.1)**	**645**	**−0.295**	**0.112**	**0.009**
Wild boar distribution index	Grade I (0.415)	516	-	-	-
	**Grade II (0.504)**	**517**	**0.216**	**0.125**	**0.086**
	Grade III (0.628)	514	−0.070	0.125	0.576
	**Grade IV (0.761)**	**517**	**−0.675**	**0.127**	**>0.001**
	**Grade V (1)**	**514**	**0.794**	**0.128**	**>0.001**
Travel time to major cities	Grade I (57)	647	-	-	-
	Grade II (107.5)	642	−0.048	0.112	0.670
	**Grade III (213)**	**645**	**−0.252**	**0.112**	**0.025**
	Grade IV (860)	644	0.146	0.111	0.191
Soil moisture	Grade I (137.6)	775	-	-	-
	**Grade II (139.6)**	**975**	**−0.670**	**0.098**	**>0.001**
	**Grade III (141.3)**	**258**	**−0.280**	**0.144**	**0.053**
	**Grade IV (143.2)**	**570**	**−0.965**	**0.114**	**>0.001**
Temperature seasonality (bioclim 4)	Grade I (9726.3)	645	-	-	-
	**Grade II (10,073)**	**646**	**−0.264**	**0.112**	**0.018**
	**Grade III (10,226.8)**	**642**	**−0.221**	**0.112**	**0.048**
	**Grade IV (10,435)**	**645**	**−0.311**	**0.112**	**0.005**
Temperature annual range (bioclim 7)	Grade I (381)	656	-	-	-
	Grade II (390)	670	−0.14	0.110	0.189
	**Grade III (396)**	**650**	**−0.27**	**0.111**	**0.015**
	**Grade IV (411)**	**602**	**−0.36**	**0.114**	**0.002**
Precipitation seasonality (bioclim 15)	Grade I (81)	660	-	-	-
	Grade II (91)	641	0.099	0.111	0.374
	Grade III (98)	656	−0.062	0.111	0.573
	Grade IV (105)	621	0.039	0.112	0.728
Precipitation of warmest quarter (bioclim 18)	Grade I (731)	658	-	-	-
	**Grade II (792)**	**634**	**0.190**	**0.112**	**0.089**
	**Grade III (849)**	**651**	**0.161**	**0.111**	**0.145**
	**Grade IV (911)**	**635**	**−0.209**	**0.112**	**0.064**
Precipitation of coldest quarter (bioclim 19)	Grade I (72)	693	-	-	-
	**Grade II (78)**	**610**	**−0.247**	**0.112**	**0.027**
	Grade III (94)	640	−0.060	0.110	0.583
	Grade IV (163)	635	0.093	0.110	0.397

**Table 3:** Results of the Generalized linear logistic regression analysis. Variables with *p*-values less than 0.05 are shown in bold.

Variables	Variable	Total Samples (n = 2578)	Coefficients	Standard Errors	Odds Ratio (95% CI)	*p*-Value	VIF
Elevation	Grade I	648	-	-	-	-	1.38
	**Grade II**	**649**	**0.530**	**0.164**	**1.70 (1.24–2.35)**	**0.001**	
	**Grade III**	**640**	**1.142**	**0.195**	**3.13 (2.14–4.60)**	**>0.001**	
	**Grade IV**	**641**	**1.062**	**0.237**	**2.89 (1.82–4.61)**	**>0.001**	
Distance from road (roadway and sidewalk)	Grade I	645	-	-	-	-	1.04
	Grade II	644	−0.130	0.125	0.88 (0.69–1.12)	0.300	
	Grade III	644	−0.210	0.127	0.81 (0.63–1.04)	0.099	
	**Grade IV**	**645**	**−0.519**	**0.132**	**0.60 (0.46–0.77)**	**>0.001**	
Wild boar distribution index	Grade I	516	-	-	-	-	1.08
	Grade II	517	0.173	0.150	1.19 (0.89–1.60)	0.248	
	Grade III	514	−0.071	0.154	0.93 (0.69–1.26)	0.646	
	**Grade IV**	**517**	**−0.378**	**0.190**	**0.69 (0.47–0.99)**	**0.047**	
	**Grade V**	**514**	**0.643**	**0.261**	**1.90 (1.15–3.21)**	**0.014**	
Travel time to major cities	Grade I	647	-	-	-	-	1.10
	Grade II	642	0.137	0.132	1.15 (0.89–1.49)	0.299	
	Grade III	645	−0.004	0.144	1.00 (0.75–1.32)	0.976	
	**Grade IV**	**644**	**0.465**	**0.154**	**1.59 (1.18–2.15)**	**0.002**	
Soil moisture	Grade I	775	-	-	-	-	1.31
	**Grade II**	**975**	**−1.341**	**0.158**	**0.26 (0.19–0.36)**	**>0.001**	
	Grade III	258	−0.219	0.181	0.80 (0.56–1.15)	0.226	
	**Grade IV**	**570**	**−1.005**	**0.155**	**0.37 (0.27–0.50)**	**>0.001**	
Temperature annual range (bioclim 7)	Grade I	656	-	-	-	-	1.36
	**Grade II**	**670**	**−0.817**	**0.182**	**0.44 (0.31–0.63)**	**>0.001**	
	**Grade III**	**650**	**−1.230**	**0.224**	**0.29 (0.19–0.45)**	**>0.001**	
	**Grade IV**	**602**	**−1.068**	**0.227**	**0.34 (0.22–0.54)**	**>0.001**	
Precipitation of warmest quarter (bioclim 18)	Grade I	658	-	-	-	-	1.46
	**Grade II**	**634**	**0.561**	**0.162**	**1.75 (1.28–2.41)**	**>0.001**	
	**Grade III**	**651**	**0.420**	**0.207**	**1.52 (1.02–2.29)**	**0.042**	
	Grade IV	635	−0.107	0.260	0.90 (0.54–1.50)	0.679	
Precipitation of coldest quarter (bioclim 19)	Grade I	693	-	-	-	-	1.78
	**Grade II**	**610**	**−0.748**	**0.207**	**0.47 (0.31–0.71)**	**>0.001**	
	**Grade III**	**640**	**−1.698**	**0.266**	**0.18 (0.11–0.31)**	**>0.001**	
	**Grade IV**	**635**	**−1.834**	**0.317**	**0.16 (0.09–0.30)**	**>0.001**	

## Data Availability

Epidemiological data on wild boar applied in this study are not publicly available and were kindly provided by the Dodram pig research center, Republic of Korea.

## References

[1] Sanchez-Vizcaino J.M., Mur L., Gomez-Villamandos J.C., Carrasco L. (2015). An update on the epidemiology and pathology of African swine fever. J. Comp. Pathol..

[2] De la Torre A., Bosch J., Iglesias I., Munoz M.J., Mur L., Martinez-Lopez B., Martinez M., Sanchez-Vizcaino J.M. (2015). Assessing the risk of African swine fever introduction into the European Union by wild boar. Transbound. Emerg. Dis..

[3] Food and Agriculture Organisation of the United Nations Statistics [FAO] (2017). African Swine Fever: Detection and Diagnostic. A Manual for Veterinarians. http://www.fao.org/3/a-i7228e.pdf.

[4] Chenais E., Depner K., Guberti V., Dietze K., Viltrop A., Stahl K. (2019). Epidemiological considerations on African swine fever in Europe 2014–2018. Porc. Health Manag..

[5] Guberti V., Khomenko S., Masiulis M., Kerba S. (2019). African Swine Fever in Wild Boar Ecology and Biosecurity.

[6] Adkin A., Coburn H., England T., Hall S., Hartnett E., Marooney C., Wooldridge M., Watson E., Cooper J., Cox T. (2004). Risk Assessment for the Illegal Import of Contaminated Meat and Meat Products into Great Britain and the Subsequent Exposure of GB Livestock (IIRA): Foot and Mouth Disease (FMD), Classical Swine Fever (CSF), African Swine Fever (ASF), Swine Vesicular Disease (SVD).

[7] De Carvalho Ferreira H.C., Weesendorp E., Quak S., Stegeman J.A., Loeffen W.L. (2014). Suitability of faeces and tissue samples as a basis for non-invasive sampling for African swine fever in wild boar. Vet. Microbiol..

[8] World Organisation for Animal Health (2022). African Swine Fever. https://www.woah.org/en/disease/african-swine-fever/.

[9] Kedkovid R., Sirisereewan C., Thanawongnuwech R. (2020). Major swine viral diseases: An Asian perspective after the African swine fever introduction. Porc. Health Manag..

[10] OIE-WAHIS: World Animal Health Information System (2022). Animal Disease Events. https://wahis.oie.int/#/events?viewAll=true.

[11] Ministry of Agriculture, Food and Rural Affairs, South Korea (2022). Release of Information on Outbreaks of Livestock Infectious Diseases (ASF). https://mafra.go.kr/FMD-AI2/2241/subview.do.

[12] Ministry of Agriculture, Food and Rural Affairs, South Korea (2022). Press Release: African Swine Fever. http://www.me.go.kr/search/totalSearch/search.jsp.

[13] Jo Y.S., Gortázar C. (2021). African swine fever in wild boar: Assessing interventions in South Korea. Transbound. Emerg. Dis..

[14] Segye (2022). Detecting Dogs Are Deployed to Search for Dead Wild Boar Bodies. https://www.segye.com/newsView/20221016506928.

[15] Ministry of Environment, South Korea (2022). Detecting the Dead Body of a Wild Boar. https://me.go.kr/niwdc/web/board/read.do?menuId=24&boardId=1555430&boardMasterId=794&condition.hideCate=1.

[16] PIGPEOPLE (2022). Real-Time Status Board of African Swine Fever (ASF). http://www.pigpeople.net/mobile/article.html?no=7260.

[17] Ministry of Environment, South Korea (2019). The Habitat Density of Wild Boars Is Mainly Determined by the Capacity of the Habitat Environment. https://me.go.kr/home/web/board/read.do?boardMasterId=1&boardId=1038690&menuId=286.

[18] Handonnews (2021). ASF-Mediated, the Habitat Density of Wild Boars is Lowered. http://handonnews.kr/mobile/article.html?no=23592.

[19] PIGPEOPLE (2019). Domestic Wild Boar Expert: “It Is Impossible to Estimate the Number of Wild Boars in the Country”. http://www.pigpeople.net/news/article.html?no=7626.

[20] Dodram Pig Farmers Cooperative (2022). General Information. http://home.dodram.com/.

[21] FAO African Swine Fever in the Russian Federation: Risk Factors for Europe and Beyond Empres Watch 2013. https://www.fao.org/3/aq240e/aq240e.pdf.

[22] FAO EMPRES-i Epidemiology (Frequency of African Swine Fever in Republic of Korea). https://empres-i.apps.fao.org/.

[23] Iglesias I., Rodriguez A., Feliziani F., Rolesu S., de la Torre A. (2017). Spatio-temporal Analysis of African Swine Fever in Sardinia (2012–2014): Trends in Domestic Pigs and Wild Boar. Transbound. Emerg. Dis..

[24] ESRI (2022). How Multi-Distance Spatial Cluster Analysis (Ripley’s K-function) Works. https://desktop.arcgis.com/en/arcmap/10.3/tools/spatial-statistics-toolbox/h-how-multi-distance-spatial-cluster-analysis-ripl.htm.

[25] Kulldorff M. (2010). SaTScan.

[26] Bosch J., Barasona J.A., Cadenas-Fernandez E., Jurado C., Pintore A., Denurra D., Cherchi M., Vicente J., Sanchez-Vizcaino J.M. (2020). Retrospective spatial analysis for African swine fever in endemic areas to assess interactions between susceptible host populations. PLoS ONE.

[27] CGIAR-CSI (2004). SRTM 90m DEM Digital Elevation Database. https://srtm.csi.cgiar.org/.

[28] South Korea National Spatial Data Infrastructure Portal (2022). Data Catalog. http://www.nsdi.go.kr/lxportal/?menuno=2679.

[29] Esri Near (Analysis). https://pro.arcgis.com/ja/pro-app/latest/tool-reference/analysis/near.htm.

[30] Tucker C.J., Pinzon J.E., Brown M.E., Slayback D.A., Pak E.W., Mahoney R., Vermote E.F., El Saleous N. (2005). An extended AVHRR 8-km NDVI dataset compatible with MODIS and SPOT vegetation NDVI data. Int. J. Remote Sens..

[31] Bosch J., Mardones F., Pérez A., De la Torre A., Muñoz M.J. (2014). A maximum entropy model for predicting wild boar distribution in Spain. Span. J. Agric. Res..

[32] Bosch J., Iglesias I., Munoz M.J., de la Torre A. (2017). A Cartographic Tool for Managing African Swine Fever in Eurasia: Mapping Wild Boar Distribution Based on the Quality of Available Habitats. Transbound. Emerg. Dis..

[33] Kim E.-T., Pak S.-I. (2020). Species distribution modeling for wild boar (Sus scropa) in the Republic of Korea using MODIS data. J. Prev. Vet. Med..

[34] IFORCE (2022). Global Accessibility Map. https://forobs.jrc.ec.europa.eu/products/gam/.

[35] Unninayar S., Olsen L.M. (2015). Monitoring, Observations, and Remote Sensing—Global Dimensions. Reference Module in Earth Systems and Environmental Sciences.

[36] Willmott C.J., Kenji M. (2001). Terrestrial Water Budget Data Archive: Monthly Time Series (1950–1999). https://sage.nelson.wisc.edu/data-and-models/atlas-of-the-biosphere/mapping-the-biosphere/ecosystems/soil-moisture/.

[37] Bergmann H., Schulz K., Conraths F.J., Sauter-Louis C. (2021). A review of environmental risk factors for African Swine Fever in European wild boar. Animals.

[38] Hijmans R.J., Cameron S.E., Parra J.L., Jones P.G., Jarvis A. (2005). Very high resolution interpolated climate surfaces for global land areas. Int. J. Climatol. J. R. Meteorol. Soc..

[39] Hidano A. (2020). Introduction to Regression Analysis for Epidemiological Data (1). J. Vet. Epidemiol..

[40] Hothorn T., Zeileis A., Farebrother R.W., Cummins C., Millo G., Mitchell D., Zeileis M.A. Package ‘Lmtest’. Testing Linear Regression Models. https://cran.r-project.org/web/packages/lmtest/lmtest.pdf.

[41] Bates D.M. (2010). Lme4: Mixed-Effects Modeling with R.

[42] Fox J., Weisberg S., Adler D., Bates D., Baud-Bovy G., Ellison S., Firth D., Friendly M., Gorjanc G., Graves S. (2012). Package ‘Car’.

[43] Cessie L., Houwelingen J. (1991). A Goodness-of-fit test for binary regression models, based on smoothing methods. Biometrics.

[44] Le Cessie S., van Houwelingen J. (1995). Goodness of fit tests for generalized linear models based on random effect models. Biometrics.

[45] Jennie Pearce S.F. (2000). Evaluating the predictive performance of habitat models developed using logistic regression. Ecol. Model..

[46] Delamater P.L., Street E.J., Leslie T.F., Yang Y.T., Jacobsen K.H. (2019). Complexity of the Basic Reproduction Number (R0). Emerg. Infect. Dis..

[47] Anderson R.M., May R.M. (1992). Infectious Diseases of Humans: Dynamics and Control.

[48] Iglesias I., Perez A., Sánchez-Vizcaíno J., Muñoz M., Martínez M., De La Torre A. (2011). Reproductive ratio for the local spread of highly pathogenic avian influenza in wild bird populations of Europe, 2005–2008. Epidemiol. Infect..

[49] Iglesias I., Munoz M.J., Montes F., Perez A., Gogin A., Kolbasov D., de la Torre A. (2016). Reproductive Ratio for the Local Spread of African Swine Fever in Wild Boars in the Russian Federation. Transbound. Emerg. Dis..

[50] Marcon A., Linden A., Satran P., Gervasi V., Licoppe A., Guberti V. (2019). R_0_ estimation for the African swine fever epidemics in wild boar of Czech Republic and Belgium. Vet. Sci..

[51] Lim J.-S., Kim E., Ryu P.-D., Pak S.-I. (2021). Basic reproduction number of African swine fever in wild boars (Sus scrofa) and its spatiotemporal heterogeneity in South Korea. J. Vet. Sci..

[52] Chenais E., Ståhl K., Guberti V., Depner K. (2018). Identification of wild boar–habitat epidemiologic cycle in African swine fever epizootic. Emerg. Infect. Dis..

[53] Pietschmann J., Guinat C., Beer M., Pronin V., Tauscher K., Petrov A., Keil G., Blome S. (2015). Course and transmission characteristics of oral low-dose infection of domestic pigs and European wild boar with a Caucasian African swine fever virus isolate. Arch. Virol..

[54] Kim H.-J., Cho K.-H., Ryu J.-H., Jang M.-K., Chae H.-G., Choi J.-D., Nah J.-J., Kim Y.-J., Kang H.-E. (2020). Isolation and Genetic Characterization of African Swine Fever Virus from Domestic Pig Farms in South Korea, 2019. Viruses.

[55] Depner K., Gortazar C., Guberti V., Masiulis M., More S., Oļševskis E., Thulke H.H., Viltrop A., Woźniakowski G. (2017). Epidemiological analyses of African swine fever in the Baltic States and Poland: (Update September 2016–September 2017). EFSA J..

[56] Fischer M., Hühr J., Blome S., Conraths F.J., Probst C. (2020). Stability of African swine fever virus in carcasses of domestic pigs and wild boar experimentally infected with the ASFV “Estonia 2014” isolate. Viruses.

[57] Probst C., Gethmann J., Amendt J., Lutz L., Teifke J.P., Conraths F.J. (2020). Estimating the postmortem interval of wild boar carcasses. Vet. Sci..

[58] Lim S.J., Han S.H., Park J.Y., Kim N.H., Namgung H., Oh Y., Park Y.C. (2022). Wildlife as Potential Vectors of African Swine Fever Virus. J. For. Environ. Sci..

[59] Amendolia S., Lombardini M., Pierucci P., Meriggi A. (2019). Seasonal spatial ecology of the wild boar in a peri-urban area. Mammal Res..

[60] Ibaraki Prefecture (2020). The Ecology of Wild Boars. https://www.pref.ibaraki.jp/nourinsuisan/hokunourin/kikaku/kikaku/inoshishinoseitai.html.

[61] Cukor J., Linda R., Václavek P., Šatrán P., Mahlerová K., Vacek Z., Kunca T., Havránek F. (2020). Wild boar deathbed choice in relation to ASF: Are there any differences between positive and negative carcasses?. Prev. Vet. Med..

[62] Cukor J., Linda R., Václavek P., Mahlerová K., Šatrán P., Havránek F. (2020). Confirmed cannibalism in wild boar and its possible role in African swine fever transmission. Transbound. Emerg. Dis..

[63] Pittiglio C., Khomenko S., Beltran-Alcrudo D. (2018). Wild boar mapping using population-density statistics: From polygons to high resolution raster maps. PLoS ONE.

[64] Thurfjell H., Spong G., Ericsson G. (2014). Effects of weather, season, and daylight on female wild boar movement. Acta Theriol..

[65] Hankyung.com (2020). Yeoncheon-gun Damaged 4.5 km of Wild Boar Fences Due to Heavy Rain. https://www.hankyung.com/politics/article/202008100999Y.

[66] National Integrated Drought Information System (2022). Soil Moisture. https://www.drought.gov/topics/soil-moisture.

[67] Ministry of Agriculture, Food and Rural Affairs, South Korea The Government Actively Promotes Measures to Prevent the Spread of African Swine Fever in Wild Boar 2021. https://www.mafra.go.kr/FMD-AI2/2241/subview.do.

[68] Sun E., Huang L., Zhang X., Zhang J., Shen D., Zhang Z., Wang Z., Huo H., Wang W., Huangfu H. (2021). Genotype I African swine fever viruses emerged in domestic pigs in China and caused chronic infection. Emerg. Microbes Infect..

[69] Sun E., Zhang Z., Wang Z., He X., Zhang X., Wang L., Wang W., Huang L., Xi F., Huangfu H. (2021). Emergence and prevalence of naturally occurring lower virulent African swine fever viruses in domestic pigs in China in 2020. Sci. China Life Sci..

[70] Ito S., Bosch J., Martínez-Avilés M., Sánchez-Vizcaíno J.M. (2022). The Evolution of African Swine Fever in China: A Global Threat?. Front. Vet. Sci..

